# A Co‐Developed Evaluation Framework for a Patient Engagement Programme Within a National Dementia Research Network

**DOI:** 10.1111/hex.70899

**Published:** 2026-10-15

**Authors:** Mastoora Rizai, Linda Grossman, Inbal Itzhak, Rosette Loughlin, Karen Myers Barnett, Ellen Snowball, Juanita‐Dawne Bacsu, Kelly Beaton, Emma Lanza, Jennifer Bethell

**Affiliations:** ^1^ Dalla Lana School of Public Health University of Toronto Toronto Ontario Canada; ^2^ Canadian Consortium on Neurodegeneration in Aging (CCNA), Engagement of People with Lived Experience of Dementia (EPLED) Advisory Group Toronto Canada; ^3^ Lady Davis Institute, Centre intégré universitaire de santé et de services sociaux du Centre‐Ouest‐de‐l'Île‐de‐Montréal Montreal Québec Canada; ^4^ KITE Research Institute, Toronto Rehabilitation Institute University Health Network Toronto Ontario Canada; ^5^ School of Nursing Thompson Rivers University Kamloops British Columbia Canada

**Keywords:** dementia, evaluation, patient and public involvement, patient engagement

## Abstract

**Introduction:**

While there is growing consensus on the importance of engaging people with lived experience in research, there is little agreement on how best to evaluate these initiatives. Co‐designed frameworks tailored to specific contexts have been recommended. Our objective was to co‐develop an evaluation framework to assess the implementation and impact of the Canadian Consortium on Neurodegeneration in Aging (CCNA)'s Engagement of People with Lived Experience of Dementia (EPLED) programme.

**Methods:**

We followed a four‐step process: (1) determine objectives for engagement; (2) co‐develop a theory of change; (3) identify quantitative and qualitative methods to collect information and develop an evaluation plan; and (4) establish a feedback loop. The framework was co‐produced with four members of the EPLED Advisory Group: a person living with dementia and three caregivers/care partners.

**Results:**

The programme logic model, a visual roadmap, was a foundational step, articulating programme activities and their linkages to intended outcomes. The framework was guided by four evaluation questions, each aligned with indicators, methods and information sources. Our data collection approach includes data collection from Advisory Group members and researchers, using questionnaires and an arts‐based evaluation design tailored to promote inclusivity and engagement. Data will be collated and disseminated through various mediums and responses from the arts‐based activity will be compiled into a collective digital mural.

**Conclusion:**

Our report contributes to the existing literature by providing an innovative example of a co‐developed evaluation framework in the context of a national research network. The insights could inform future efforts in evaluating initiatives intended to meaningfully engage patient partners in research, particularly people with lived experience of dementia.

**Patient or Public Contribution:**

People with lived experience took part in all aspects of this work. They contributed to the development of the funding proposal and were listed as co‐investigators on the grant. They were involved in the development of the evaluation framework described herein and have co‐authored the current manuscript.

## 1 Introduction

Dementia is a global health concern that is expected to rise as the population ages. Primary prevention is a key strategy to address dementia; it has been estimated that addressing 14 risk factors might prevent or delay nearly half of new dementia cases [1]. While emerging disease‐modifying therapies may offer promise for some types of dementia [2], there remains a critical need for effective tertiary prevention approaches that enhance the quality of life for people living with dementia and their care partners [3].

The Canadian Consortium on Neurodegeneration in Aging (CCNA) was initiated in 2014 to advance research on neurodegenerative diseases. It is a pan‐Canadian network made up of clinicians, researchers and others working in the areas of prevention, treatment, and quality of life. CCNA is funded by the Canadian Institutes of Health Research and partner organisations, including the Alzheimer Society of Canada [4]. CCNA researchers are supported by programmes, including Engagement of People with Lived Experience of Dementia (EPLED). EPLED was formed as a part of CCNA in Phase II (2019‐2024). Its primary activity is to support an Advisory Group, composed of people with lived experience (i.e., people living with dementia, caregivers/care partners). Advisory Group members were recruited through online platforms (e.g., social media), Alzheimer Societies, Strategy for Patient Oriented Research (SPOR) support units in provinces across Canada, and other networks [5].

While there is growing agreement on the importance of engaging people with lived experience in research, there is little consensus and clarity on the best approaches to evaluating these initiatives. Without evaluation, efforts to assess the impact of patient engagement and improve practice are limited, and may risk having negative effects, for example, power imbalances, failure to recognise different ways of knowing, and experiences of tokenism. Multiple frameworks [6] and evaluation tools exist [7, 8, 9, 10] and can be used to support patient engagement in research. Although many evaluation themes, such as context, activities, people, processes, and outcomes, may apply across initiatives, frameworks should be co‐designed and adapted to the specific contexts in which they will be used [6, 7, 10]. Co‐development is essential as it enables researchers and community members to collaboratively shape the framework, ensuring it meets needs while promoting trust and a shared sense of ownership [11].

For the EPLED programme, considerations for designing evaluation activities include the imperative of participatory evaluation [12], the area of research (i.e., dementia), and the setting in which the research takes place (i.e., a national network of multiple teams, spanning research activities and types of research, including biomedical, clinical, population health, quality of life, and health services). Evaluation of patient engagement in research, more generally, has highlighted challenges in adapting evaluation tools to be responsive, inclusive, and relevant to specific contexts [13]. Existing evaluation frameworks, developed more broadly for health research, may be limited in their applicability in this context. More specifically, they do not necessarily incorporate the values and preferences of people with lived experience of dementia, who may have unique challenges (e.g., accessibility) [14] and have varied motivations for engaging in research, including efforts to address stigmas associated with dementia [15]. Further, their approach to defining people with lived experience may not be nuanced for the context of dementia, where the experiences of persons living with dementia can be closely intertwined with those of their caregivers and care partners. While their perspectives should be considered as distinct [16], persons living with dementia and their caregivers or care partners are often engaged together and this can both enable and limit expression [17].

The objective of this paper is to describe the co‐development of a tailored evaluation framework for the EPLED programme, including a logic model and multiple methods of data collection in an arts‐based evaluation component. We aim to provide practical guidance that can support others seeking to evaluate initiatives designed to meaningfully engage people with lived experience in research.

## 2 Materials and Methods

The project team included the EPLED lead researcher (JBe), EPLED staff (ES, EL), CCNA staff (II), and EPLED Advisory Group members (LG, RL, KMB, and an individual who opted not to participate as a co‐author). The authorship team also added individuals involved in CCNA phase III, including the new EPLED co‐lead researcher (JBa) and an Advisory Group member (KB), who contributed to the preparation of this manuscript. For the project team, Advisory Group members (one individual living with dementia and three caregivers/care partners) were involved at the outset as co‐investigators on a University Health Network (UHN) Pride in Patient Engagement in Research (PiPER) internal grant application. The four Advisory Group members had been involved with EPLED since the Advisory Group's inception in 2020 and the work benefitted from both their knowledge and expertise as well as the trusting relationships they had developed among themselves and the broader team. Given that Advisory Group members were geographically distributed across Canada, the group used Zoom as a virtual meeting platform for regular meetings to accommodate members' varying schedules and time zones [18]. Building on members' familiarity and comfort with each other and with virtual collaboration platforms, all meeting activities related to the development of the evaluation framework were conducted online through Zoom.

A Master of Public Health practicum student (MR) was recruited from the Dalla Lana School of Public Health to execute the co‐development of the EPLED evaluation framework from May until August 2024. We reviewed existing resources (i.e., frameworks, logic models, metrics, evaluation tools) and then worked collaboratively to formulate an approach tailored to the context of CCNA and EPLED. We mainly focused on resources that discussed the involvement of patients, people with lived experience and community members in research and those that highlighted enhancing transparency and accountability in these collaborations. We considered outcomes both within and outside the research collaboration [7], quantitative and qualitative reporting, as well as tracking over time and across the programme lifecycle [19]. In designing the evaluation framework, we mirrored steps outlined by Vat et al, [10] namely: (1) Determine objectives for engagement; (2) Co‐develop a theory of change; (3) Identify data collection methods and develop and evaluation plan; and (4) Establish a feedback loop. The detail of each step is explained below.

### 2.1 Determine Objectives for Engagement

At the time of the project, CCNA had just entered Phase III, a 5‐year funding period from 2024 to 2029, alongside the introduction of a new model of operations whereby EPLED would be one of the components of the CCNA Operations Centre's Dementia Research Support Hub. The objectives for EPLED remained largely unchanged from CCNA Phase II, that is, to: (1) Support persons with dementia and care partners to be actively involved in research; (2) Work with CCNA researchers, programmes, and partners to develop novel mechanisms and formats to further this collaboration; and (3) Advance the methods of patient engagement in research by embedding evaluation processes that will measure the impact of these initiatives. Throughout Phase III, EPLED's focus also includes supporting newly funded research teams addressing diverse topics related to dementia (e.g., biomarkers, cardiovascular disease and age‐related cognitive changes and health equity in caregiving).

### 2.2 Co‐Develop a Theory of Change

To enable active engagement in the co‐development process, the practicum student planned and facilitated information sessions for the project team to build their capacity, confidence, and knowledge about public health programme planning and evaluation. Initially, the student prepared and delivered a 1‐h virtual session to introduce the concepts of logic models and programme theory. This was followed by a second 1‐h virtual session that explored the purpose and value of logic models and shared examples [20, 21] related to patient engagement in research. The session also included a facilitated discussion to identify priorities and vision for the EPLED logic model. Insights from these sessions and iterative refinement shaped the co‐development of the EPLED logic model, depicting the programme's activities and their linkages to intended results (i.e., with inputs, activities, outputs, outcomes, and impacts). Between May and June 2024, the practicum student led this process and refined the details of the programme theory and logic model. Session time was used to build the Advisory Group member's understanding of public health programme planning, enabling meaningful feedback and active contributions to discussions on programme priorities and vision that informed the theory of change. The resulting logic model provided a shared understanding of EPLED's work and served as an important foundation for the evaluation framework.

### 2.3 Identify Quantitative and Qualitative Methods to Collect Information and Develop an Evaluation Plan

Using the co‐produced logic model as the basis, we co‐developed the EPLED evaluation framework. First, the practicum student prepared and delivered a 1‐h virtual session to familiarise the project team with the concepts of programme evaluation, approaches to collecting evaluation data, and data analysis. This session also included discussions on the evaluability of EPLED and refinement of the evaluation plan's objectives and scope. Second, the practicum student reviewed existing evaluation tools identified from systematic reviews [7, 8, 9, 10]. These resources were also used to identify approaches for incorporating co‐design into the evaluation framework development process. The practicum student prepared an evaluation draft using these existing tools, which was presented during the second virtual session to gather feedback from the project team. The draft outlined potential evaluation questions, indicators to measure the programme's intended outcomes, and proposed methods for how these would be measured. Feedback provided during the session helped refine the EPLED evaluation framework. A revised draft was then shared with the team to ensure that key points and ideas from the discussions were adequately captured. Advisory Group members were also given the chance to provide feedback via Zoom instead of submitting written feedback, if preferred. Questions, indicators, methods and sources were reported according to an adapted “concept‐indicator‐method” approach [22] and whereby indicators were classified according to the metrics framework proposed by Vat et al. [10] The concept‐indicator‐method approach was useful as it clearly linked key evaluation concepts in an easy‐to‐follow way for the team. It guided us in systematically structuring the evaluation to ensure it generated relevant information aligned with our evaluation objectives. The approach began with identifying core *concepts* derived from programme objectives. For each concept, specific *indicators* were selected to describe the concept. Once the concepts and indicators were defined, the next step was to determine the appropriate *methods* and *sources* for gathering the required information. When introducing the approach to the project team, examples were used to help illustrate how it works in practice.

### 2.4 Establish a Feedback Loop for the Advisory Group, CCNA and Its Partners

In preparing the manuscript, the team discussed and agreed on the approach for collating, analysing, and reporting the information to reach academic and non‐academic audiences. The goal of our manuscript was to provide guidance to future patient engagement evaluation efforts by sharing our process and learnings with the wider community. We also anticipated approaches to sharing the evaluation framework through community‐focused presentations to reach non‐academic audiences and disseminating the evaluation findings to programme funders, partners, and Advisory Group members.

## 3 Results

We determined that the objective of the EPLED evaluation framework was to assess the implementation and impacts of the programme, with the aim of improving approaches to programme activities conducted in CCNA Phase III (2024‐2029). The evaluation activities would be conducted and managed by the EPLED programme, including any associated expenses, and were designed to be practical and within EPLED's existing resources. An overview of the contributions from the project team during the development of the evaluation framework is outlined in Table 1.

**Table 1:** Overview of contributions from Advisory Group members and other members of the project team in developing the Engagement of People with Lived Experience of Dementia (EPLED) evaluation framework.

Stage of EPLED evaluation framework development	Advisory Group member contributions	Researcher and staff contributions	Student contributions
Preparation and planning	Co‐developed the funding proposal and co‐investigators on the grant.	Co‐developed the funding proposal and co‐investigators on the grant.	None.
Determine objectives for engagement	None. Engagement objectives were already established.	None. Engagement objectives were already established.	None. Engagement objectives were already established.
Co‐develop a theory of change	Reviewed each component of the logic model and provided feedback and refinements.	Reviewed each component of the logic model and provided feedback and refinements.	Organised and delivered educational sessions to build knowledge of public health programme planning and evaluation skills; shared examples of logic models; developed an initial draft of the EPLED logic model; and refined the EPLED logic model based on team feedback.
Identify quantitative and qualitative methods to collect information and develop an evaluation plan	Shared ideas to inform draft evaluation questions, methods and measures; reviewed, refined and proposed new (i.e., arts‐based) approaches to evaluation; and provided input on the accessibility and feasibility of data collection.	Shared ideas to inform draft evaluation questions, methods and measures; reviewed, refined and proposed new (i.e., arts‐based) approaches to evaluation; reviewed academic literature; and obtained institutional review approval.	Organised and delivered educational sessions; moderated brainstorming discussions; developed an initial draft of evaluation questions, methods and measures; and refined evaluation plan based on team feedback.
Establish a feedback loop for the Advisory Group, CCNA and its partners	Identified academic and non‐academic audiences (in addition to EPLED programme) and approaches to sharing knowledge.	Identified academic and non‐academic audiences (in addition to EPLED programme) and approaches to sharing knowledge.	None.

### 3.1 Determine Objectives for Engagement

The objectives of EPLED were determined at the outset, see above, and maintained unaltered throughout this process.

### 3.2 Co‐Develop a Theory of Change

We co‐developed the EPLED logic model (see Figure 1) that delineates inputs, activities, outputs, outcomes, and impacts. We intentionally aligned the impacts with Canada's National Dementia Strategy, which identifies three national objectives: preventing dementia, advancing therapies and finding a cure, and improving the quality of life for people living with dementia and their caregivers [3]. The strategy also outlines four to five areas of focus under each objective; for example, access to quality care is a key focus under the third objective. While we recognised the broad scope of these long‐term impacts, we considered it important to define and articulate EPLED's ultimate purpose: to make a difference for individuals impacted by dementia. As a visual roadmap, the logic model describes the resources required to carry out programme activities, the direct outputs generated and how these contribute to achieving the desired short‐ and long‐term outcomes, ultimately driving programme impacts. We have no expectation of evaluating every step in the logic model, however, we aimed to create and communicate a shared understanding of EPLED's role in contributing to lasting changes for individuals and society.

**Figure 1:**
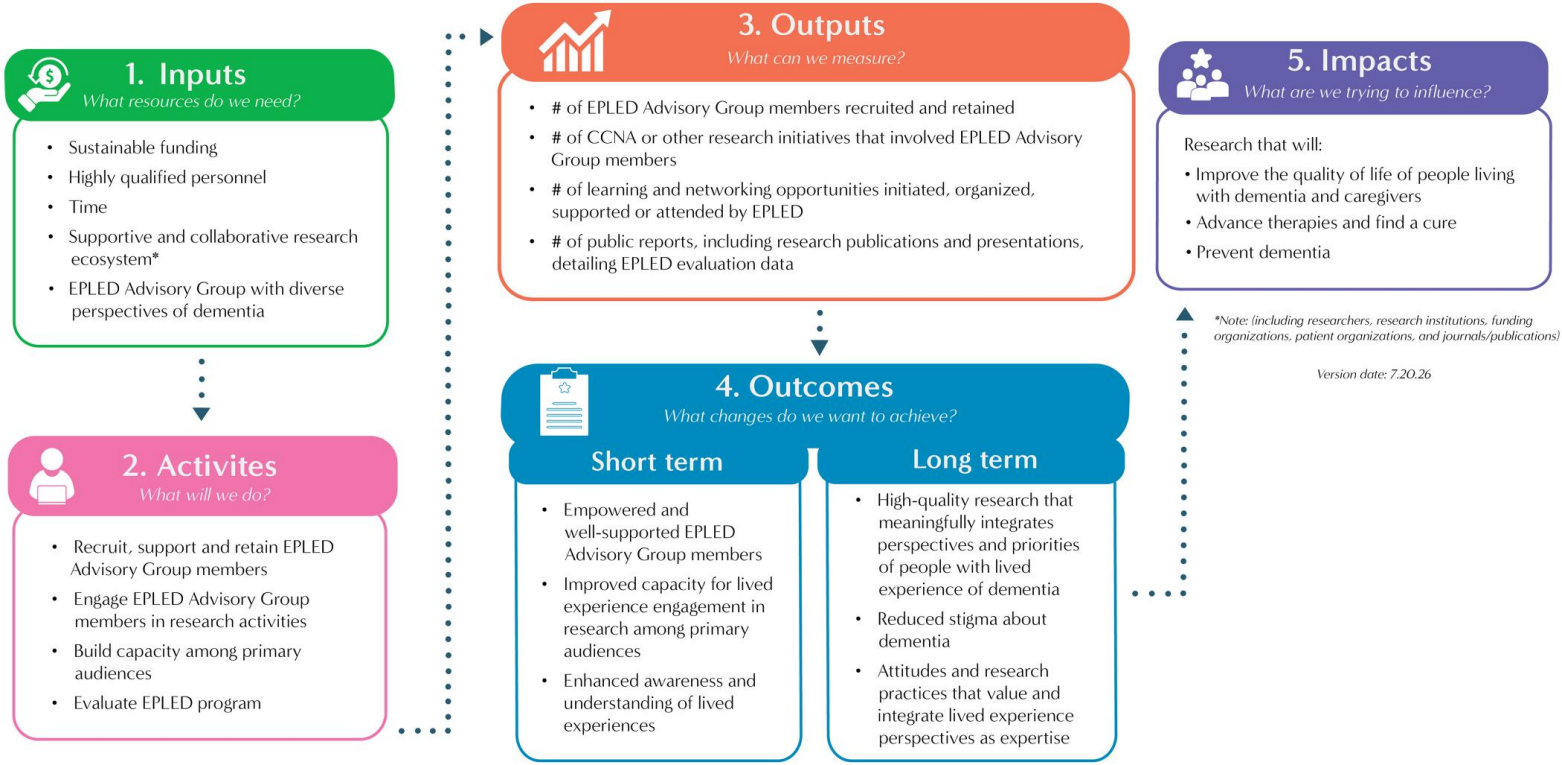
Canadian Consortium on Neurodegeneration in Aging (CCNA) Engagement of People with Lived Experience of Dementia (EPLED) Logic Model.

### 3.3 Identify Quantitative and Qualitative Methods to Collect Information and Develop an Evaluation Plan

We identified four questions to guide the evaluation, and aligned indicators, methods and information sources accordingly (see Table 2). These questions were selected because the team was interested in exploring these aspects and they also reflected programme's objectives. The first question focused on the programme's effort to have diverse representation. The second question assessed how programme activities are perceived by Advisory Group members and CCNA researchers, in terms of promoting a safe and collaborative environment, meeting their expectations, and responding to feedback. The third question evaluated the effectiveness of the programme in successfully integrating perspectives and experiences of people with lived experience of dementia in research. Finally, the fourth question explored the broader benefits of the programme for both Advisory Group members and the CCNA research community.

**Table 2:** Matrix of evaluation questions, indicators, methods and sources identified for Engagement of People with Lived Experience of Dementia (EPLED) evaluation framework.

Evaluation question	How will we answer this question? (indicators)	How would we measure or observe this? (methods)*					Who will provide the data? (sources)	
		1	2	3	4	5	Advisory Group members	Researchers (including trainees and programme staff)
1. Was the EPLED programme successful in recruiting a diverse Advisory Group membership?	Diversity of lived experience representatives (i.e., Advisory Group members)	✓	✓	✓			✓	✓
2. What are the views and experiences of the EPLED programme from the perspectives of Advisory Group members and CCNA researchers (including trainees and programme staff)?	Clarity of the roles in the patient engagement activity		✓				✓	
	Opportunity to contribute to patient engagement activities		✓				✓	
	Feelings of trust, honesty, transparency, respect, shared learning and/or a give‐and‐take relationship		✓				✓	
	Satisfaction with support from activity organisers		✓	✓			✓	✓
	Satisfaction with the patient engagement activity		✓	✓			✓	✓
	Willingness to continue the collaboration		✓	✓			✓	✓
	Individual changes and impacts				✓		✓	
3. Was the EPLED programme effective in engaging people with lived experience of dementia in research?	Number, type and frequency of patient engagement activity					✓		✓
	Influence of lived experience insights on research decision making		✓	✓			✓	✓
	Change in awareness and knowledge about needs of people with lived experience			✓				✓
4. Was the EPLED programme effective in improving capacity for patient engagement in research among EPLED Advisory Group members and researchers (including trainees and programme staff)?	Learnings from patient engagement activity/activities		✓	✓			✓	✓

*Methods: 1. Advisory Group Onboarding Survey; 2. Advisory Group Annual Survey; 3. Researcher End‐of‐Project Survey; 4. Advisory Group Exit Activity; 5. Research project descriptive data.

We designed five forms of data collection to occur at multiple time points, which are summarised in Table 3 and provided in Appendix A–E. Throughout the co‐development process, Advisory Group members highlighted the importance of accessibility, including the use of plain language, familiar on‐line platforms, support when needed, minimising participant burden, and employing methods that would facilitate self‐expression. Although we considered collecting interview data, we felt that practical considerations such as time and expertise requirements would exceed the resources available, precluding this form of data collection. We incorporated open‐ended and closed‐ended questions in our surveys and included an arts‐based activity. We intentionally included trainees and programme staff in data collection from researchers, recognising their important roles in facilitating and conducting research.

**Table 3:** Summary of data sources identified for Engagement of People with Lived Experience of Dementia (EPLED) evaluation framework.

Methods	Brief description	Who/what will provide the data? (sources)		Frequency and timing
		Advisory Group members	Researchers (including trainees and programme staff)	
1. Advisory Group Onboarding Survey	Questionnaire; demographic characteristics (13 closed‐ended questions)	✓		One‐time, on joining Advisory Group
2. Advisory Group Annual Survey	Questionnaire; perspectives and experiences of EPLED programme activities (10 closed‐ended questions and 2 open‐ended questions)	✓		Repeated annually and on term completion or departure from Advisory Group
3. Researcher End‐of‐Project Survey	Questionnaire; perspectives and experiences of EPLED programme activities (10 closed‐ended questions and 2 open‐ended questions)		✓	One‐time, on completion of EPLED‐involved project
4. Advisory Group Exit Activity	Arts‐based activity; perspectives and experiences of EPLED programme activities	✓		One‐time, on term completion or departure from Advisory Group
5. Research project descriptive data	On‐line submission completed when requesting EPLED involvement; project details (8 project attributes) and number of Advisory Group members involved		✓	One‐time, when requesting EPLED input then supplemented by EPLED (number of Advisory Group members involved)

#### 3.3.1 Questionnaires

In alignment with guidance from the University Health Network, we first devised the Advisory Group Onboarding Survey as a “minimum dataset” of sociodemographic information to be collected from Advisory Group members to capture the diversity of the group and contextualise evaluation findings [23]. To gain multiple perspectives, we then developed the Advisory Group Annual Survey and Researcher End‐of‐Project Survey.

To improve the accessibility of the questionnaires for both Advisory Group members and researchers, we limited: the number of questions overall (maximum: 15); the number of open‐ended/free text questions (maximum: 2); and, for closed‐ended questions, the number of response options (maximum: 5, except when asking about race [24]), and included an explicit “I prefer not to answer” option for all questions. For Advisory Group members, we chose to use icons alongside text with some multiple‐choice options, and an explicit statement that the questionnaire could be completed independently or with help from others, including assistance from EPLED staff if required. Questionnaire data will be collected by EPLED staff through REDCap, which is an on‐line, encrypted platform. All data will be collected without identifiers and never linked to an individual during analysis and reporting.

#### 3.3.2 Arts‐Based Activity

To promote inclusivity, facilitate meaningful engagement, and minimise participant burden, we selected an arts‐based evaluation approach to complement the Advisory Group surveys. While members can provide feedback through open‐ended survey questions, the arts‐based approach will offer an additional opportunity, through creative expression, to reflect on and communicate how the EPLED programme has impacted them. This approach honours the diverse forms of contribution, particularly for members whose cognitive abilities may shift over time. It affirms that their wisdom and insights remain invaluable, even as their communication styles or capacities evolve. As part of the broader evaluation framework, the arts‐based approach created an engaging, inclusive, and accessible way to gain insight into Advisory Group members' experiences of the EPLED programme [25, 26, 27].

Using a collage‐making approach, EPLED Advisory Group members will be given instructions to respond to the prompt, “*How did the EPLED programme change or impact you?*”. To create the individual collages, Advisory Group members will be encouraged to work with materials that are comfortable and accessible to them, including old magazines and books, photographs, illustrations, poems, etc. For members who do not have these materials readily available, we will offer to send an art supply kit via mail. Acknowledging the vulnerability of making and sharing art [26], Advisory Group members will be offered the opportunity to work independently by submitting their pieces via email or collaboratively by joining a 90‐min virtual group art‐making session, facilitated by an EPLED programme manager with a background in fine art (ES). Further, for the latter, EPLED staff will also participate in the art‐making session. To support expression of meaning and avoid mis‐/over‐interpretation, participants will also be invited to provide a brief one‐ or two‐sentence explanation of their art, either via email or during the group session [26]. To assemble the individual experiences into a broader EPLED narrative, with consent from the participant, the EPLED programme manager will compile the images into a larger, digital group mural.

#### 3.3.3 Research Project Descriptive Data

Research project descriptive data will be recorded from researchers (including trainees and programme staff) when requesting EPLED collaboration. This data is collected through the EPLED website, and a subset will be used to describe research project activities. We will also track the proportion of CCNA teams that collaborate with EPLED and the number of EPLED collaboration requests that are fulfilled.

These evaluation methods were reviewed and approved by the University Health Network Quality Improvement Review Committee (QIRC 25‐1051) who also provided a Research Ethics Board exemption.

### 3.4 Establish a Feedback Loop for the Advisory Group, CCNA and Its Partners

EPLED staff will collate evaluation data into spreadsheets, recording the number and proportion of participants who complete each data collection activity. EPLED staff will quantify and describe responses to closed‐ended questions and research project descriptive data as frequencies and proportions, using charts and tables to improve visualisation of the data. No formal statistical testing or comparisons (e.g., cross‐tabulations) are planned. EPLED staff will identify themes from open‐ended questions.

The results will be disseminated in a variety of venues, which will include both academic and non‐academic audiences. In addition to this academic article, we will share our evaluation framework through an infographic, a co‐presented webinar and conference presentations across EPLED, CCNA, University Health Network's Pride in Patient Engagement in Research (PiPER) initiative, and other networks involving researchers and people with lived experience.

Evaluation data will support understanding of the programme's effectiveness and impact while also informing programme decision‐making and guiding ongoing improvement efforts. In particular, data will be reviewed in EPLED's annual reporting to identify actionable feedback that will enable the programme to make changes in response to identified issues or concerns. Over the longer term, the information will be used to develop any future EPLED programmes beyond the current CCNA phase III.

## 4 Discussion

We co‐developed an evaluation framework to assess the implementation and impact of the EPLED programme within a national research network. We did this by adapting existing resources and considering multiple approaches that would be applicable to our setting and useful for our programme activities. We worked together to develop multiple approaches to data collection, mindful of feasibility and acceptability of any primary data collection from Advisory Group members and researchers, with an emphasis on balancing comprehensiveness with brevity. For Advisory Group members, in particular, we included both questionnaires and an arts‐based evaluation activity as a strategy for fostering inclusivity (i.e., accessibility) and encouraging self‐reflection and ‐expression. Our co‐developed logic model served as both the precursor to the evaluation as well as a communication tool for discussing EPLED more broadly.

Our evaluation framework includes both standard (i.e., survey) and less conventional (i.e., arts‐based) forms of data collection, which is characteristic of a participatory evaluation approach [12]. We selected an arts‐based approach to create an opportunity to share experiences, through creative expression, and address some of the potential barriers to participation [25]. As this framework represents EPLED's first effort to systematically incorporate evaluation, our priority was to encourage member participation while minimising burden. We considered additional sources of qualitative data (e.g., interviews and focus groups) to gather insights, however, practical concerns (e.g., team time and capacity for analysis) as well as potential influences of bias and power dynamics narrowed our focus. By incorporating creative expression, we sought opportunities for EPLED members to share experiences of unintended effects and those that fall outside of our predetermined EPLED objectives and associated logic model [26]. Our approach mirrors other co‐designed evaluation frameworks involving older adults; although unknown to us at the time of developing our framework, Abousifein and colleagues described a similar co‐design process with older adults, including incorporating an arts‐based approach (photovoice) to encourage creative expression and a novel approach to sharing findings [28]. A co‐developed evaluation framework, specific to engagement in the context of pharmaceutical development, similarly highlighted the value of qualitative and quantitative data, noting the difficulty of quantifying learnings from engagement activities [19].

Our evaluation framework makes a contribution to the research literature for both the evaluation framework as well as the process for co‐developing it, building on existing guidance. Our work was built on the EPLED programme's foundational activities, with sustained research funding, through CCNA Phase II (2019‐2024). More specifically, our project benefitted from collective knowledge and established relationships, developed through the 5‐year collaboration (i.e., it involved Advisory Group members and others associated with EPLED from its inception). EPLED Advisory Group members had previously collaborated, with each other and with other members of the project team, in similar ways on other projects. Through the EPLED programme, they were supported in these roles based on their own needs and interests. These aspects were foundational to the project. Through its composition, our team was mindful of perspectives and tailoring evaluation tools to researchers and people with lived experience. Recognising the importance of multiple perspectives, we intentionally included both Advisory Group and researcher experiences on specific aspects of engagement, such as the impact of lived experience insights on research decision making and the diversity of lived experience representatives. These two perspectives may have some discordance and, in particular, it has been shown that researchers report more engagement across all phases of research than their patient partners [29].

By reporting on the co‐development process, we hope others working in similar contexts will adapt it for their own purposes. Yet, we acknowledge limitations to our framework and approach. We have laid out our plans and expectations, however, the reality of data collection will present challenges. We anticipate that there may be issues with the acceptability or feasibility of both the Advisory Group and researcher data collection ultimately creating problems of non‐response and missing data. While we recognise our need to consider the impacts of missing data in interpreting our findings, we also recognise that implementing our evaluation framework will require the same approaches as we have maintained with the programme itself, including developing relationships, providing education, offering support and being flexible in our approach [18]. Indeed, participatory evaluation is grounded in these trusting relationships and opportunities for mutual learning [12]. For our approach, we followed steps described by Vat et al, [10] however, other approaches have also been described. The Patient‐Centred Outcomes Research Institute (PCORI) described 28 concepts within 5 domains: engagement context, engagement activities, engagement quality, partner outcomes and (near‐term and long‐term) research outcomes [30]. Our evaluation framework does not include research outcomes that are more project specific (e.g., patient‐centredness of study) or assessed at the community level (e.g., changes in policy and practice or health outcomes); although incorporated into our EPLED logic model, and important as observable impacts [8], these are more difficult to assess than outputs. These, as well as assessments of group dynamics (e.g., solidarity, mutual support, and cohesion [7]), which were considered critical for early EPLED activities, are potential areas for further development. Finally, our logic model presents simplified, linear relationships; in reality, EPLED operates within a broader context that we have only minimally acknowledged (i.e., with mention of a supportive and collaborative research ecosystem). These contextual factors will influence EPLED programme outcomes, and a deeper understanding of the programme context [31], in particular, would offer valuable insights for patient engagement programmes more broadly.

## 5 Conclusion

Evaluation is critical in advancing the methods of patient engagement. Participatory evaluation methods, including co‐design of evaluation frameworks, must be central to these activities. We offer an approach for co‐developing an evaluation framework for a national dementia research network, with potential to adapt to other research‐related contexts. In doing so, we highlight the importance of incorporating methods to draw on diverse perspectives and conceptualising evaluation as an opportunity for mutual capacity building.

## Author Contributions

**Mastoora Rizai:** conceptualisation, writing – original draft, methodology, writing – review and editing, project administration, resources. **Linda Grossman:** writing – review and editing. **Inbal Itzhak:** writing – review and editing. **Rosette Loughlin:** writing – review and editing. **Karen Myers Barnett:** writing – review and editing. **Ellen Snowball:** writing – review and editing, visualisation, conceptualisation. **Juanita‐Dawne Bacsu:** writing – review and editing. **Kelly Beaton:** writing – review and editing. **Emma Lanza:** writing – review and editing. **Jennifer Bethell:** conceptualisation, supervision, writing – original draft, methodology, resources, funding acquisition.

## Conflicts of Interest

The authors declare no conflicts of interest.

## Data Availability

The authors have nothing to report.

## Appendix A EPLED Advisory Group Onboarding Survey

Hello,

You are being invited to complete a UHN Quality Improvement (QI) **EPLED Advisory Group Onboarding Survey** because you are a member of the Engagement of People with Lived Experience of Dementia (EPLED) Advisory Group. By completing this survey, you will help us to better understand the diversity of EPLED Advisory Group members. The data you provide will be used to create academic and non‐academic knowledge products (e.g., manuscripts, videos, infographics).

**Privacy:**

Please note that taking part in this survey is optional and the information is collected without identifiers and never linked to an individual. If you decide not to participate, your care or employment will not be affected in any way. All information will be de‐identified in analysis and reporting. It will be seen by EPLED staff («insert names») and stored on a password protected, encrypted UHN server for 10 years. Do not include any information that could identify you in any of your answers (e.g., your email, name, address, etc.). Whether on‐site or remotely, UHN makes all efforts to ensure that your information is shared in a way that is secure and private (encrypted). However, any electronic communication carries some risk of third parties gaining unauthorised access to information.

**Instructions:**

This survey should be completed electronically using the following URL link. Complete the survey fields and click “submit” at the bottom of the page. You will receive a confirmation email after you have successfully submitted your answers.

**Link:** «insert redcap link»

**Please note that this link should NOT be shared*.

If you have questions about your rights as a participant in a UHN Quality Improvement Project, please contact the UHN Quality Improvement Review Committee (QIRC) at <<insert email address>>. QIRC is a group of people who oversee the ethical conduct of QI projects; they are not part of the project team.

*If you require assistance, please contact EPLED programme manager* «*insert name*»

*(EPLED Phone #* «*insert UHN telephone number*», «*insert UHN email address*»).

Thank you

[Diversity of lived experience representatives (i.e., Advisory Group members)]

1. What perspective do you bring to the EPLED programme? [select all that apply]
  a. Person living with dementia
  b. Friend, family, caregiver or care partner
  c. Other, please specify: _______
2. What type(s) of dementia do you have lived experience of? [select all that apply]
  a. Alzheimer's disease
  b. Vascular dementia
  c. Frontotemporal dementia
  d. Dementia with Lewy bodies
  e. Other, please specify: _______
  f. I prefer not to answer
3. Do you have any previous experience contributing to research as a person with lived experience? [select only one]
  a. Yes
  b. No
  c. I prefer not to answer
4. How old are you? [select only one]
  a. Under 25 years
  b. 25 to 44 years
  c. 45 to 64 years
  d. 65 to 84 years
  e. 85 years and older
  f. I prefer not to answer
5. Were you born in Canada? [select only one]
  a. Yes
  b. No
  c. I prefer not to answer
6. Which term(s) best describe your current gender identity? [select all that apply]
  a. Man
  b. Woman
  c. Non‐binary person
  d. A gender not specified above, please specify: _______
  e. I prefer not to answer
7. Please select the sexual orientation that best describes how you currently think of yourself. [select only one]
  a. Straight (heterosexual)
  b. 2SLGBTQ+
  c. I don't identify with any option provided. I identify as: ______
  d. I prefer not to answer
8. Which category(ies) best describes you? [select all that apply]
  a. Black (African, African Canadian, Afro‐Caribbean descent)
  b. East Asian (Chinese, Japanese, Korean, Taiwanese descent)
  c. Indigenous (First Nations, Inuk/Inuit, Métis)
  d. Latin American (Hispanic or Latin American descent)
  e. Middle Eastern (Arab, Persian, West Asian descent (e.g., Afghan, Egyptian, Iranian, Kurdish, Lebanese, Turkish)
  f. South Asian (South Asian descent e.g., Bangladeshi, Indian, Indo‐Caribbean, Pakistani, Sri‐Lankan)
  g. White (European descent)
  h. Another race category; please specify ________
  i. Do not know
  j. I prefer not to answer
9. What is the highest level of education that you have completed? [select only one]
  a. Less than secondary (high) school graduation
  b. Secondary (high) school diploma or equivalent
  c. Some postsecondary education (highest)
  d. Postsecondary certificate, diploma or degree
  e. I prefer not to answer
10. Are you currently working at a job or business (e.g., employee, self‐employed or both)? [select only one]
  a. Yes
  b. No
  c. I prefer not to answer
11. Which province of Canada do you live in? [select only one]
  a. Alberta
  b. British Columbia
  c. Ontario
  d. Newfoundland and Labrador
  e. Prince Edward Island
  f. Nova Scotia
  g. New Brunswick
  h. Quebec
  i. Manitoba
  j. Saskatchewan
  k. Yukon
  l. Northwest Territories
  m. Nunavut
  n. I prefer not to answer
12. What type of community do you live in? [select only one]
  a. Rural area
  b. Small city or town (population < 100,000)
  c. City (population 100,000‐499,000)
  d. Large city (population > 500,000)
  e. I prefer not to answer
13. What language do you speak most often at home? [select only one]
  a. English
  b. French
  c. Another language
  d. I prefer not to answer

## Appendix B EPLED Advisory Group Annual Survey

Hello,

You are being invited to complete a UHN Quality Improvement (QI) **EPLED Advisory Group Annual Survey** because you are a member of the Engagement of People with Lived Experience of Dementia (EPLED) Advisory Group. By completing this survey, you will help us understand and improve experiences of the EPLED Advisory Group and programme. The data you provide will be used to create academic and non‐academic knowledge products (e.g., manuscripts, videos, infographics).

**Privacy:**

Please note that taking part in this survey is optional and the information is collected without identifiers and never linked to an individual. If you decide not to participate, your care or employment will not be affected in any way. All information will be de‐identified in analysis and reporting. It will be seen by EPLED staff («insert names») and stored on a password protected, encrypted UHN server for 10 years. Do not include any information that could identify you in any of your answers (e.g., your email, name, address, etc.). Whether on‐site or remotely, UHN makes all efforts to ensure that your information is shared in a way that is secure and private (encrypted). However, any electronic communication carries some risk of third parties gaining unauthorised access to information.

**Instructions:**

This survey should be completed electronically using the following URL link. Complete

The survey fields and click “submit” at the bottom of the page. You will receive a

Confirmation email after you have successfully submitted your answers.

**Link:** «insert redcap link»

**Please note that this link should NOT be shared*.

If you have questions about your rights as a participant in a UHN Quality Improvement Project, please contact the UHN Quality Improvement Review Committee (QIRC) at <<insert email address>>. QIRC is a group of people who oversee the ethical conduct of QI projects; they are not part of the project team.

*If you require assistance, please contact EPLED programme manager* «*insert name*»

*(EPLED Phone* # «*insert UHN telephone number*», «*insert UHN email address*»).

Thank you

1. What perspective do you bring to the EPLED programme? (check all that apply)
  a. Person living with dementia
  b. Friend, family, caregiver or care partner
  c. Other, please specify: _______
2. I have a clear understanding of the purpose of the EPLED programme.
  [Clarity of the roles in the patient engagement activity]
3. I have the information and support that I need to participate in the EPLED programme activities (e.g., travel reimbursement, technical or learning support, meeting reminders, etc.)
  [Satisfaction with support from activity organisers]
4. I am able to express my perspectives in my work with the EPLED programme.
  [Opportunity to contribute to patient engagement]
5. My thoughts and perspectives are heard, valued and received by the EPLED programme.
  [Feelings of trust, honesty, transparency, respect, shared learning and/or a give‐and‐take relationship]
6. The individuals participating in the EPLED programme represent a broad range of perspectives.
  [Diversity of lived experience representatives (i.e., Advisory Group members)]
7. I have made an impact through my involvement with the EPLED programme.
  [Influence of lived experience insights on research decision making]
8. The experience of working with the EPLED programme has been valuable and meaningful to me.
  [Satisfaction with the patient engagement activity]
9. The EPLED programme is achieving its stated objectives.
  [Influence of lived experience insights on research decision making]
10. The experience of working with the EPLED programme has helped me to learn about research and dementia.
  [Learnings from patient engagement activity/activities]
11. How do you think we could improve the EPLED programme? [space for open‐ended response]

What impacts do you think the EPLED programme has had? This could include impact on you, the research or more broadly. [space for open‐ended response].

## Appendix C EPLED Researcher End‐of‐Project Survey

Hello,

You are being invited to complete a UHN Quality Improvement (QI) **EPLED Researcher End‐of‐Project Survey** because you have collaborated with the Engagement of People with Lived Experience of Dementia (EPLED) Advisory Group. By completing this survey, you will help us understand and improve experiences of the EPLED Advisory Group and programme. The data you provide will be used to create academic and non‐academic knowledge products (e.g., manuscripts, videos, infographics).

**Privacy:**

Please note that taking part in this survey is optional and the information is collected without identifiers and never linked to an individual. If you decide not to participate, your care or employment will not be affected in any way. All information will be de‐identified in analysis and reporting. It will be seen by EPLED staff («insert names») and stored on a password protected, encrypted UHN server for 10 years. Do not include any information that could identify you in any of your answers (e.g., your email, name, address, etc.). Whether on‐site or remotely, UHN makes all efforts to ensure that your information is shared in a way that is secure and private (encrypted). However, any electronic communication carries some risk of third parties gaining unauthorised access to information.

**Instructions:**

This survey should be completed electronically using the following URL link. Complete the survey fields and click “submit” at the bottom of the page. You will receive a confirmation email after you have successfully submitted your answers.

**Link:** «insert redcap link»

**Please note that this link should NOT be shared*.

If you have questions about your rights as a participant in a UHN Quality Improvement Project, please contact the UHN Quality Improvement Review Committee (QIRC) at <<insert email address>>. QIRC is a group of people who oversee the ethical conduct of QI projects; they are not part of the project team.

*If you require assistance, please contact EPLED programme manager* «*insert name*»

*(EPLED Phone* #«*insert UHN telephone number*», «*insert UHN email address*»).

Thank you

1. What is your role in research?
  a. CCNA Researcher
  b. CCNA Trainee
  c. CCNA research staff
  d. Not CCNA affiliated, please specify: _______
  e. Other, please specify: _______
2. What is your primary research theme? (Check only one)
  - Biomedical
  - Clinical
  - Health systems/services
  - Social/cultural/environmental/population health
  - Not applicable/specified

3. The perspectives of those who will be affected by my research were represented through the EPLED Advisory Group members.
  [Diversity of lived experience representatives (i.e., Advisory Group members)]
  a. Strongly disagree
  b. Disagree
  c. Neither agree nor disagree
  d. Agree
  e. Strongly agree
4. I was satisfied with the support I received from the EPLED programme.
  [Satisfaction with support from activity organisers]
  a. Strongly disagree
  b. Disagree
  c. Neither agree nor disagree
  d. Agree
  e. Strongly agree
5. Involvement with the EPLED programme was a good use of our project resources.
  [Satisfaction with the patient engagement activity]
  a. Strongly disagree
  b. Disagree
  c. Neither agree nor disagree
  d. Agree
  e. Strongly agree
6. The perspectives and views of EPLED Advisory Group members had a positive influence on the project.
  [Influence of lived experience insights on research decision making]
  a. Strongly disagree
  b. Disagree
  c. Neither agree nor disagree
  d. Agree
  e. Strongly agree
7. As a result of my involvement with the EPLED programme, I am better informed about patient engagement in research and perspectives of people with lived experience of dementia.
  [Change in awareness and knowledge about needs of people with lived experience]
  a. Strongly disagree
  b. Disagree
  c. Neither agree nor disagree
  d. Agree
  e. Strongly agree
8. As a result of my involvement with the EPLED programme, I am comfortable leading future patient engagement activities.
  [Learnings from patient engagement activity/activities]
  a. Strongly disagree
  b. Disagree
  c. Neither agree nor disagree
  d. Agree
  e. Strongly agree
9. I would consider opportunities to work with the EPLED programme in the future.
  [Willingness to continue the collaboration]
  a. Strongly disagree
  b. Disagree
  c. Neither agree nor disagree
  d. Agree
  e. Strongly agree
10. The EPLED programme is achieving its stated objectives.
  [Influence of lived experience insights on research decision making]
  a. Strongly disagree
  b. Disagree
  c. Neither agree nor disagree
  d. Agree
  e. Strongly agree
11. How do you think we could improve the EPLED programme? [space for open‐ended response]
12. What impacts do you think the EPLED programme has had? This could include impact on you, the research or more broadly. [space for open‐ended response]

## Appendix D EPLED Advisory Group Exit Activity

Hello,

You are being invited to complete a UHN Quality Improvement (QI) **EPLED End‐of‐Term Art Activity** because you are a member of the Engagement of People with Lived Experience of Dementia (EPLED) Advisory Group. By completing this survey, you will help us understand and improve experiences of the EPLED Advisory Group and programme. We are using art to collect perspectives about the EPLED programme. We ask individuals to complete a collage that will be used in a larger, digital group mural. The data you provide will be used to create academic and non‐academic knowledge products (e.g., manuscripts, videos, infographics).

Digital mural from EPLED 1.0

**Privacy:**

Please note that taking part in this survey is optional and the information is collected without identifiers and never linked to an individual. If you decide not to participate, your care or employment will not be affected in any way. All information will be de‐identified in analysis and reporting. It will be seen by EPLED staff («insert names») and stored on a password protected, encrypted UHN server for 10 years. Do not include any information that could identify you in any of your answers (e.g., your email, name, address, etc.). Whether on‐site or remotely, UHN makes all efforts to ensure that your information is shared in a way that is secure and private (encrypted). However, any electronic communication carries some risk of third parties gaining unauthorised access to information.

**Instructions:**

1. Reflect on the question: “How did the EPLED programme change or impact you?”
2. Use your own materials to create a collage to be included in a larger, digital group mural.
3. Scan, photograph or send an image of your collage to «insert email address».
4. Provide a 1‐2 sentence description of your artwork (OPTIONAL).

**Materials:** Scissors, magazines, glue or tape, wrapping paper, any other material you can collage with.

*If you require assistance, please contact EPLED programme manager*, «*insert name*»

*(EPLED Phone* # «*insert UHN telephone number*», «*insert UHN email address*»).

Thank you.

## Appendix E Project Descriptive Data

These data represent a subset of the information collected from researchers when request EPLED involvement, see: https://www.epled.ca/en/consulting

*[Number, type and frequency of patient engagement activity]*

1. Primary research theme of project:

- Biomedical
- Clinical
- Health systems/services
- Social/cultural/environmental/population health
- Not applicable/specified

2. CCNA research team project:

- Yes (CCNA Phase III)
- Yes (CCNA Phase II)
- No

3. CCNA affiliated researcher(s) as Principal Investigator or Co‐investigator:

- Yes (CCNA Phase III)
- Yes (CCNA Phase II)
- No

4. Profiles of EPLED members invited to be involved in project:

- People with dementia
- Friends/family/caregivers/care partners of people with dementia
- Both

5. Composition of Advisory Group members invited to be involved in project:

- Entire Advisory Group
- Only some members of the Advisory Group
- Not sure

6. Proposed stages of EPLED involvement in project:

- Governance (e.g., contributing to study oversight)
- Priority setting (e.g., helping to refine research questions)
- Study design and procedures (e.g., selecting outcomes, reviewing consent procedures and study documents)
- Study recruitment (e.g., suggesting avenues to recruit study participants)
- Data collection (e.g., conducting interviews)
- Data analysis (e.g., interpreting findings)
- Knowledge translation (e.g., coauthoring publications or presentations)
- Other – please specify:

7. Proposed goal of EPLED involvement in project:

- INFORM: to give information, listen and answer questions
- CONSULT: to seek input and feedback
- INVOLVE: to include people with lived experience as standing members of an advisory group
- COLLABORATE: to partner with people with lived experience as equal team members
- EMPOWER: to follow the lead of people with lived experience and support their decisions

8. Budgeted compensation for EPLED Advisory Group:

- Yes
- No
